# Fluorine-Doped Tin Oxide Colloidal Nanocrystals

**DOI:** 10.3390/nano10050863

**Published:** 2020-04-30

**Authors:** Owen Kendall, Pierce Wainer, Steven Barrow, Joel van Embden, Enrico Della Gaspera

**Affiliations:** School of Science, RMIT University, Melbourne, VIC 3000, Australia; s3609854@student.rmit.edu.au (O.K.); s3726974@student.rmit.edu.au (P.W.); steven.barrow@rmit.edu.au (S.B.); joel.vanembden@rmit.edu.au (J.v.E.)

**Keywords:** colloids, doping, metal oxides, surface plasmon resonance, FTO

## Abstract

Fluorine-doped tin oxide (FTO) is one of the most studied and established materials for transparent electrode applications. However, the syntheses for FTO nanocrystals are currently very limited, especially for stable and well-dispersed colloids. Here, we present the synthesis and detailed characterization of FTO nanocrystals using a colloidal heat-up reaction. High-quality SnO_2_ quantum dots are synthesized with a tuneable fluorine amount up to ~10% atomic, and their structural, morphological and optical properties are fully characterized. These colloids show composition-dependent optical properties, including the rise of a dopant-induced surface plasmon resonance in the near infrared.

## 1. Introduction

Plasmonic semiconducting nanocrystals (NCs), and specifically doped metal oxides have recently emerged as materials able to bridge the optoelectronic gap between metal and dielectric NCs, providing optical transparency in the visible spectral range and tuneable localized surface plasmon resonance (LSPR) peaks in the infrared [1,2]. Coatings deposited from such NCs typically show significantly enhanced conductivity compared to assemblies of their undoped counterparts. This tuneability in optical and electrical properties is related to the amount of free carriers induced by aliovalent doping, which can be modulated during NC synthesis through appropriate reaction conditions.

Thus far, the majority of research on plasmonic oxide NCs has focussed on indium tin oxide (ITO), due to the robust and reproducible protocols available for the synthesis of In_2_O_3_, and to its amenability to doping with tin [3,4,5,6,7,8]. Additionally, doped indium oxide NCs can show rather sharp plasmonic peaks at high energies, leading to NCs with high quality factor LSPR [9,10]. Research on materials alternative to ITO has seen constant increase. Doped ZnO has been extensively investigated as a cheaper, earth-abundant option, with synthetic protocols now available to synthesize ZnO NCs with a range of dopant types and LSPR energies [11,12,13,14,15,16]. Additional research has also been conducted on other plasmonic oxides including TiO_2_, WO_3_, BaSnO_3_, CdO, Ga_2_FeO_4_, highlighting the breadth and potential of this research field [17,18,19,20,21].

Considering that ITO is one of the two industry standards for transparent conducting oxide (TCO) coatings, it is not surprising that the majority of the work is done on ITO NCs. However, fluorine-doped tin oxide (FTO) is the market competitor for TCO applications, and yet surprisingly very little is available in the literature for SnO_2_-based NCs, especially with plasmonic properties. In fact, excluding a few early works on antimony-doped SnO_2_ (that show a distinctive blue color and IR absorption properties [22,23]), little work has been done on the synthesis of colloidal SnO_2_ NCs, and specifically on aliovalently doped SnO_2_ showing plasmonic properties. The few existing works include solvothermal or microwave-based reactions, which are mostly focused on antimony doping [24,25], and also some reports on FTO NCs [26,27]. However, these methods for FTO NCs usually produce powders and/or suspensions of aggregated NCs, which are not ideal for optical and optoelectronic applications.

Here, we present the colloidal synthesis of FTO NCs in the 2–4 nm size range, and analyze their properties in relation to the amount of fluorine dopant. We demonstrate that fluorine quenches the defect-related blue luminescence typical of SnO_2_, and also that the aliovalent doping imparts plasmonic resonances in the near infrared, which are a clear indication of the generation of free carriers. 

## 2. Materials and Methods 

### 2.1. Materials

Tin bis-acetylacetonate dichloride (98%), ammonium fluoride (99.99%), diphenyl ether (99%), 1,2-dodecanediol (90%) and oleylamine (70%) were purchased from Sigma Aldrich (Castle Hill, NSW, Australia). Methanol (99.8%), ethanol (99.9%), toluene (99.5%) and chloroform (99.8%) were supplied by Thermo Fisher Scientific/Univar (Scoresby, VIC, Australia). All chemicals were used without further purification.

### 2.2. Nanocrystal Synthesis

In a typical synthesis, 7.5 mL diphenyl ether, 0.8 mL oleylamine, 1 mmol 1,2-dodecanediol and 1 mmol of the tin precursor (or tin + dopant precursors) are loaded into a 50 mL three neck round bottom flask (RBF). The RBF was connected to a Schlenk line and placed under vacuum (~4 × 10^−3^ mbar) at 60 °C with the aid of a heating mantle until the precursors were fully dissolved and completely degassed (~15 min). The RBF was then backfilled with nitrogen and then heated to 240 °C within ~30 min, with the reaction being held at this temperature for 3 h. At the end of the reaction, the heating mantle was switched off, and the NC suspension was let cool naturally under a nitrogen blanket. Excess ethanol (~30 mL) is added to flocculate the NCs, which are then centrifuged (4400 rpm, 6 min) and resuspended in toluene. In some instances, methanol (5–10 mL in addition to ethanol) was required to induce precipitation of the NCs. This process is repeated 2–3 times to further purify the NCs. After the last washing, the precipitate is split, with half of the product being re-suspended and stocked in chloroform and the other half being collected and dried at 80 °C overnight obtaining a fine powder. Selected powder samples were annealed at 600 °C for 2 h in a muffle furnace. To produce more material, this protocol can be easily scaled up doubling the amount of reagents, and keeping all the other reaction parameters constant. The samples have been labeled SnO_2_ (for undoped NCs) or FTO*X*, where *X* is the doping level defined as:

*X*(%) = *n*_F_/(*n*_Sn_ + *n*_F_) × 100 (1)

where *n* is the number of moles. This notation is conventionally used for doping at the cation site, with the dopant replacing the metal ion (tin) within the oxide lattice. Here, fluorine act as a dopant at the anion site, replacing oxygen. However, for consistency with existing literature, we decided to keep this notation to define the doping level.

### 2.3. Characterization Techniques

X-ray diffraction (XRD) patterns of powder samples were collected using a Bruker D4 Endeavor diffractometer (Bruker, Billerica, MA, USA) equipped with a Cu-Kα radiation source and operated at 40 kV and 35 mA. The crystallite size was evaluated with the Scherrer relationship using the full width at half-maximum of the main diffraction peaks fitted using Lorentzian functions. Energy dispersive X-ray (EDX) spectra were acquired on a Nova 200 NanoSEM (FEI, Hillsboro, OR, USA) with a voltage of 20 kV. The fluorine doping level for each sample was averaged on at least five independent measurements. Transmission electron microscopy (TEM) images were acquired on a JEOL 2100F microscope (JEOL, Tokyo, Japan) operated at 200 kV. Optical absorption spectra in the UV and visible regions of NCs dispersed in chloroform were acquired with a Cary 60 UV-Vis spectrophotometer (Agilent, Santa Clara, CA, USA). Photoluminescence (PL) spectra of the same solutions were acquired with a Horiba Jobin Yvon Fluoromax-4 fluorometer (Horiba, Kyoto, Japan) with excitation wavelengths of 260 and 330 nm. Optical absorption spectra in the visible and NIR regions were acquired with a Cary 7000 UV-Vis-NIR spectrophotometer (Agilent, Santa Clara, CA, USA) equipped with an integrating sphere and a centermount sample holder. Fourier transform infrared (FTIR) spectroscopy was carried out using a Perkin-Elmer Frontier spectrometer (Perkin Elmer, Waltham, MA, USA) equipped with a Pike GladiATR attenuated total reflectance (ATR) stage.

## 3. Results and Discussion

FTO NCs are synthesized adapting an existing protocol developed for SnO_2_ NCs [28]. Briefly, tin (IV) bis-acetylacetonate dichloride and the desired amount of ammonium fluoride (0%, 5%, 10% or 20% nominal) are dissolved in a mixture of diphenyl ether, oleylamine and 1,2-dodecanediol. After degassing at 60 °C, the mixture is heated to 240 °C under a nitrogen atmosphere and held at that temperature for 3 h. The reaction proceeds via alcoholysis between the diol and acetylacetonate groups, generating water in-situ. This triggers the formation of SnO_2_ clusters via condensation of hydroxylated tin precursors in the presence of oleylamine as surface ligand [29]. After the completion of the reaction, the solution is cooled to room temperature and the NCs are collected and purified by means of conventional centrifugation/resuspension cycles.

**Figure 1:**
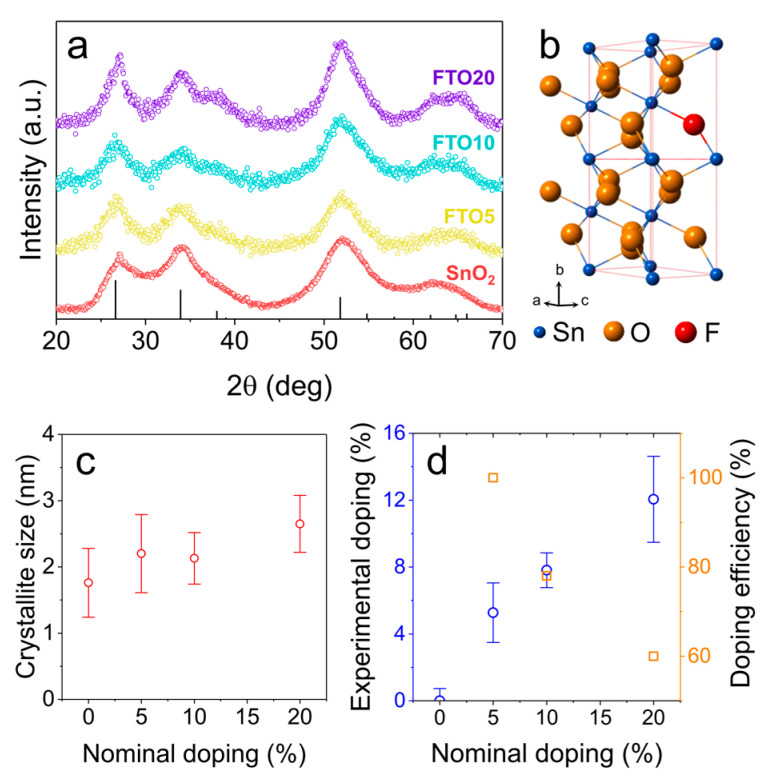
(**a**) X-ray diffraction (XRD) patterns for the undoped SnO_2_ and F-doped SnO_2_ (FTO) nanocrystals (NCs) prepared in this study. The expected diffraction peak positions for cassiterite tin dioxide are reported at the bottom. (**b**) Schematic representation of the SnO_2_ lattice. (**c**) Crystallite size as a function of the doping level. (**d**) Experimental F doping and average doping efficiency as a function of the nominal doping level.

Figure 1 shows the X-ray diffraction (XRD) patterns for undoped SnO_2_ NCs, and for FTO NCs with different nominal doping levels (5, 10 and 20 atomic% with respect to Sn, hereafter labelled FTO5, FTO10 and FTO20, respectively). Broad diffraction peaks can be seen in all samples, indicative of nanometer-size crystals. All peaks can be indexed to tetragonal cassiterite SnO_2_ (Figure 1b, ICDD No. 41-1445) without the presence of impurities. The diffraction peaks do not seem to shift with increased doping level, suggesting minimal perturbation in the crystalline lattice caused by the presence of fluoride anions. However, the broad peaks make the identification of shifts quite challenging, especially because nano-sized crystals can show deviation from the expected lattice parameter values even without the presence of extrinsic dopants [30]. Scherrer analysis was carried out to estimate the crystallite size from the full width at half maximum (FWHM) of the three main diffraction peaks: (110) at 26.6°, (101) at 33.9° and (211) at 51.8°. The average size of the crystals is seen to increase slightly (from ~1.8 nm to ~2.7 nm) when larger amounts of ammonium fluoride are added to the reaction medium. Notably, the error (standard deviation) associated with these estimates is rather large, and therefore the size increase is almost statistically insignificant (Figure 1c). Although minor, this effect might point out to a role of ammonium fluoride in controlling the nucleation and growth of SnO_2_ NCs. Interestingly, if the dried NCs are annealed at high temperature (600 °C), the expected thermally-induced grain growth is less pronounced in highly doped FTO compared to both undoped SnO_2_ and FTO samples with lower dopant concentrations. This evidence shows that once inside the SnO_2_ lattice, fluorine dopants act as defects resulting in enough lattice strain to hinder the overall grain growth of the NCs (see Figure S1 in the Supplementary).

Compositional analysis on the NC powders was carried out using energy dispersive X-ray (EDX) spectroscopy; despite fluorine being a light element, its presence could be clearly detected in the doped samples (see Figure S2 for EDX spectra), with its signal progressively increasing in samples with a higher nominal doping (Figure 1d). By comparing the experimentally observed fluorine amount with the nominal fluorine doping level (amount of ammonium fluoride used in the reaction) we can evaluate the average “doping efficiency” of our system. We achieved almost stoichiometric incorporation of fluorine at 5% nominal doping (experimental value: 5.2 ± 1.7%), and reasonably high doping levels for higher fluorine concentrations (7.8 ± 1.0% for FTO10 and 12.1 ± 1.2% for FTO20). These values correspond to average doping efficiencies of 100% for FTO5, 78% for FTO10 and 60% for FTO20. The doping efficiency is high at low doping levels, and progressively decreases at high doping levels (Figure 1d). This is due to the stresses (both physical and Coulombic) induced by the aliovalent dopants, which provide a barrier to the continual incorporation of additional dopant ions [11,12].

**Figure 2:**
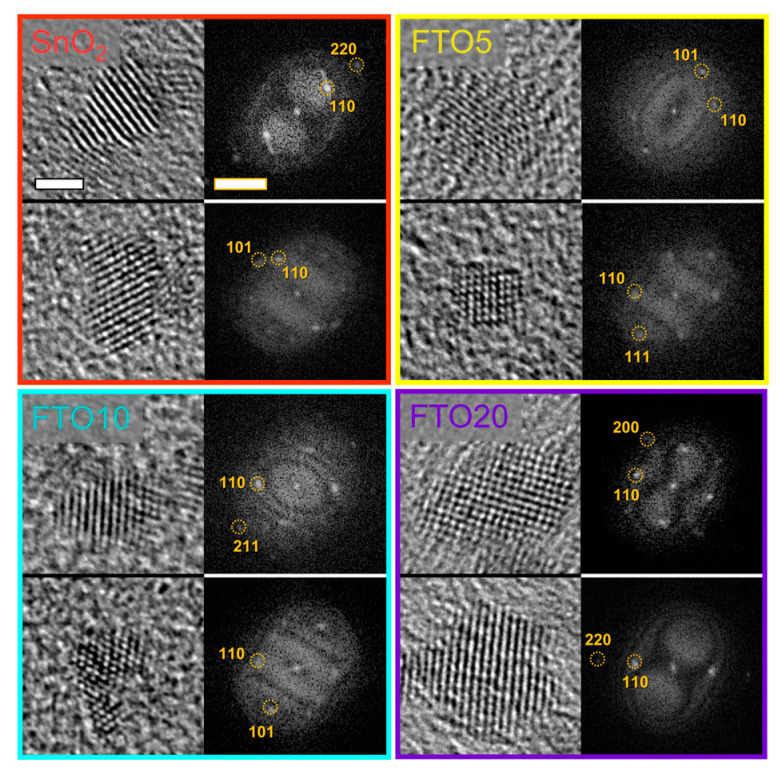
High resolution transmission electron microscopy (TEM) images of doped and undoped SnO_2_ NCs. For each sample, two images of individual NCs and the respective fast Fourier transform (FFT) images are presented. The scale bars are 2 nm for the TEM images and 2 × 1/nm for the FFT images.

Having analyzed the structural and compositional properties of our FTO NCs, we now focus on the size and the morphology of the colloids. Transmission electron microscopy (TEM) results are reported in Figure 2 and Figure S3. Small NCs, with size ranging between 2 and 4 nm can be readily seen in all samples. These values are in reasonable agreement with the crystallite size evaluated from the diffraction patterns. The NCs are slightly polydisperse and irregular in size, however, they are highly crystalline as evidenced by the high-resolution images showing distinctive lattice fringes. The respective fast Fourier transform (FFT) data show clear spots in reciprocal space that can be readily indexed to cassiterite SnO_2_, with predominant (110) and (101) lattice planes visible. No discernible variation in lattice spacing in doped samples can be observed, which is not surprising considering the extremely small size of the NCs.

**Figure 3:**
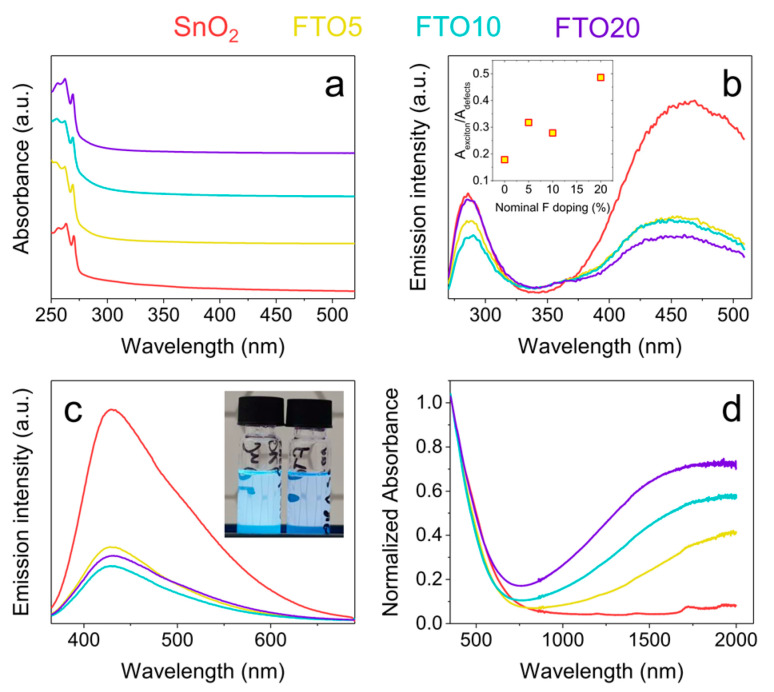
Optical characterization of SnO_2_ and FTO NCs. (**a**) Optical absorption spectra in the UV-Vis region. The spectra are vertically offset for clarity. (**b**) Photoluminescence spectra with excitation at 260 nm. The inset shows the integrated ratio between the excitonic peak and the defect peak. (**c**) Photoluminescence spectra with excitation at 330 nm. The inset shows a digital photograph of SnO_2_ (left) and FTO10 (right) colloids under UV illumination. (**d**) Absorption spectra in the UV-Vis-NIR showing a plasmon peak of increasing intensity.

The FTO NCs are synthesized in high-boiling point solvents in the presence of aliphatic ligands (oleylamine). Such ligands are still present at the surface of the NCs after purification (as demonstrated by FTIR spectroscopy, see Figure S4) and make them readily soluble in non-polar solvents without aggregation. This enables precise optical measurements to be conducted to elucidate the optical band gap and the emission properties. The optical characterization of the FTO colloids dispersed in chloroform is reported in Figure 3. All NCs show a sharp absorption onset at ~275 nm, corresponding to a band gap energy of ~4.5 eV (Figure 3a and Figure S5 for Tauc analysis). This value is larger than the band gap of bulk SnO_2_ (3.6 eV [31]), and can be readily ascribed to quantum confinement given the small size of the NCs [28,32]. Interestingly, clear excitonic transitions can be observed in the spectra, indicating the high quality and the high crystallinity of the as-prepared NCs. In addition, an absorption tail extending from the band edge towards longer wavelengths can be seen, suggesting the presence of low-energy defect states located within the band gap.

Photoluminescence (PL) spectra have been acquired to investigate the emission properties of the FTO NCs. Using an excitation wavelength of 260 nm (4.77 eV, higher energy than band gap), two emission peaks can be identified in all samples. The higher energy emission peak at ~285 nm is associated with band-edge recombination. The lower energy peak is centered in the blue spectral region and is much broader than the high energy one. This peak is due to defect-related emission, including defects such as oxygen vacancies, tin interstitials and partially reduced tin (Sn^2+^ at Sn^4+^ sites), which create additional levels within the SnO_2_ band gap [28,32,33]. This phenomenon is also commonly observed in other un-passivated quantum dots such as ZnO, ZnS and CdS [34,35,36]. Interestingly, the presence of fluorine dopants drastically decreases the intensity of this defect band, as shown in the inset of Figure 3b, where the areal (integrated) ratio between the excitonic peak and the defect peak is plotted as a function of fluorine doping. This ratio increases almost three-fold from ~0.18 for the undoped sample to ~0.49 for the FTO20 sample, showing the inverse relationship between the two peaks with the addition of fluorine. It is important to note that, while dopants can act as defects and cause an increase in defect-related emissions, they can also act as luminescence quenchers through non-radiative mechanisms, which is assumed to be the case of fluorine within SnO_2_ [36,37].

Notably, our FTO NCs can also be excited below the band gap, as shown in Figure 3c; following excitation at 330 nm (3.75 eV), all samples are seen to emit in the blue spectral region, again with the undoped SnO_2_ NCs being much brighter than the FTO NCs. This is evident in the inset of Figure 3c, which shows a digital picture of two colloidal solutions (SnO_2_, left; FTO10, right) exposed to UV illumination in ambient light. Additional photos of the two solutions under different light conditions can be found in Figure S6. The ability to excite directly sub-band gap states in SnO_2_ colloids has been previously demonstrated for undoped SnO_2_, and relies on the presence of the aforementioned defect states [28].

One of the main features that is commonly observed in TCO NCs is the presence of a LSPR peak in the near-mid infrared region following aliovalent doping. Our FTO NCs show a steady increase in the intensity of the plasmon peak in the IR with increased doping, while the undoped NCs are transparent across most of the visible and NIR spectral range (Figure 3d). This LSPR peak is a further evidence of the successful fluorine incorporation within SnO_2_, and the subsequent generation of additional charge carriers. Interestingly, the presence of a LSPR peak in such small NCs (2–4 nm), suggests that this resonance can be generated and sustained by just a few conduction band electrons per NC, as previously shown also for photodoped ZnO quantum dots [38]. Moreover, the absorption tail extending from the band gap into the visible range is observed again in all samples, which is consistent with the presence of in-gap states.

Importantly, our optimized synthetic method is applicable to the synthesis of antimony-doped tin oxide (ATO), by simply replacing the fluorine precursor with antimony chloride. ATO NCs of similar size (2–3 nm from Scherrer analysis) are obtained, and their NIR absorption properties confirm the presence of a LSPR, indicative of free carriers generated by the aliovalent dopant, in this instance at the cation site (Figure S7). These preliminary results demonstrate the applicability of this reaction to other doped SnO_2_ NCs, highlighting its potential.

## 4. Conclusions

In conclusion, we have presented a method to synthesize colloidal SnO_2_ NCs doped with fluorine, and investigated their structural, morphological and optical properties. Fluorine dopants can be incorporated within SnO_2_ in high concentration without detrimental effects on the host crystal. Their presence is further validated by the generation of a surface plasmon resonance in the near infrared associated with the increased number of free carriers. This work will stimulate and foster future studies on SnO_2_-based colloidal NCs for optoelectronic and plasmonic applications.

## Supplementary Materials

The following are available online at https://www.mdpi.com/2079-4991/10/5/863/s1, Figure S1: Additional XRD. Figure S2: EDX characterization. Figure S3: Additional TEM. Figure S4: FTIR spectra. Figure S5: Tauc plots. Figure S6: Digital photo of colloidal solutions. Figure S7: Characterization of Sb-doped SnO_2_ nanocrystals.
