# Supplementary material for: Fluorine-Doped Tin Oxide Colloidal Nanocrystals
Source: Nanomaterials (Basel). 2020 Apr 30;10(5):863. doi: 10.3390/nano10050863 (PMC7712819; doi:10.3390/nano10050863)
Supplement: Supplementary file 1 [file nanomaterials-10-00863-s001.pdf]

*Supporting Information*

# Fluorine-Doped Tin Oxide Colloidal Nanocrystals

**Owen Kendall [1], Pierce Wainer [1], Steven J. Barrow [1], Joel van Embden [1], and Enrico Della Gaspera [1,*]**

[1] School of Science, RMIT University, Melbourne, VIC 3000, Australia; s3609854@student.rmit.edu.au (O.K.); s3726974@student.rmit.edu.au (P.W.); steven.barrow@rmit.edu.au (S.J.B.); joel.vanembden@rmit.edu.au (J.V.E.)

* Correspondence: enrico.dellagaspera@rmit.edu.au

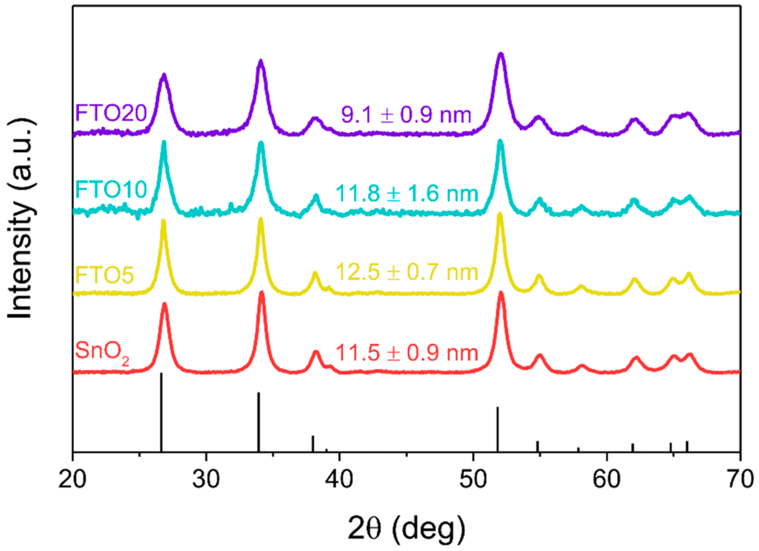


**Figure S1.** Diffraction (XRD) patterns for $SnO_2$ and FTO NC powders annealed at 600 °C in air for 2 hours. The crystallite size estimated with the Scherrer relationship is also reported.

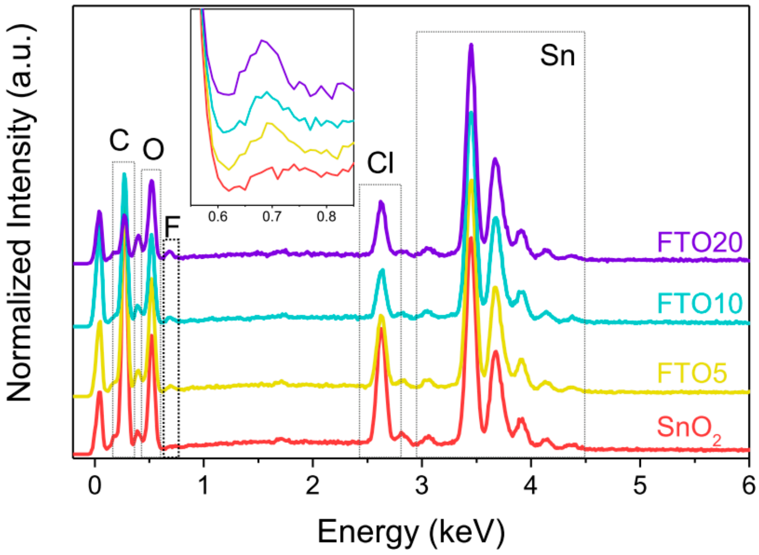


**Figure S2.** Dispersive X-ray (EDX) spectra for $SnO_2$ and FTO NCs. The intensity has been normalized to the tin signal, and the spectra are vertically offset for clarity. The inset shows a zoomed view of the fluorine peak.

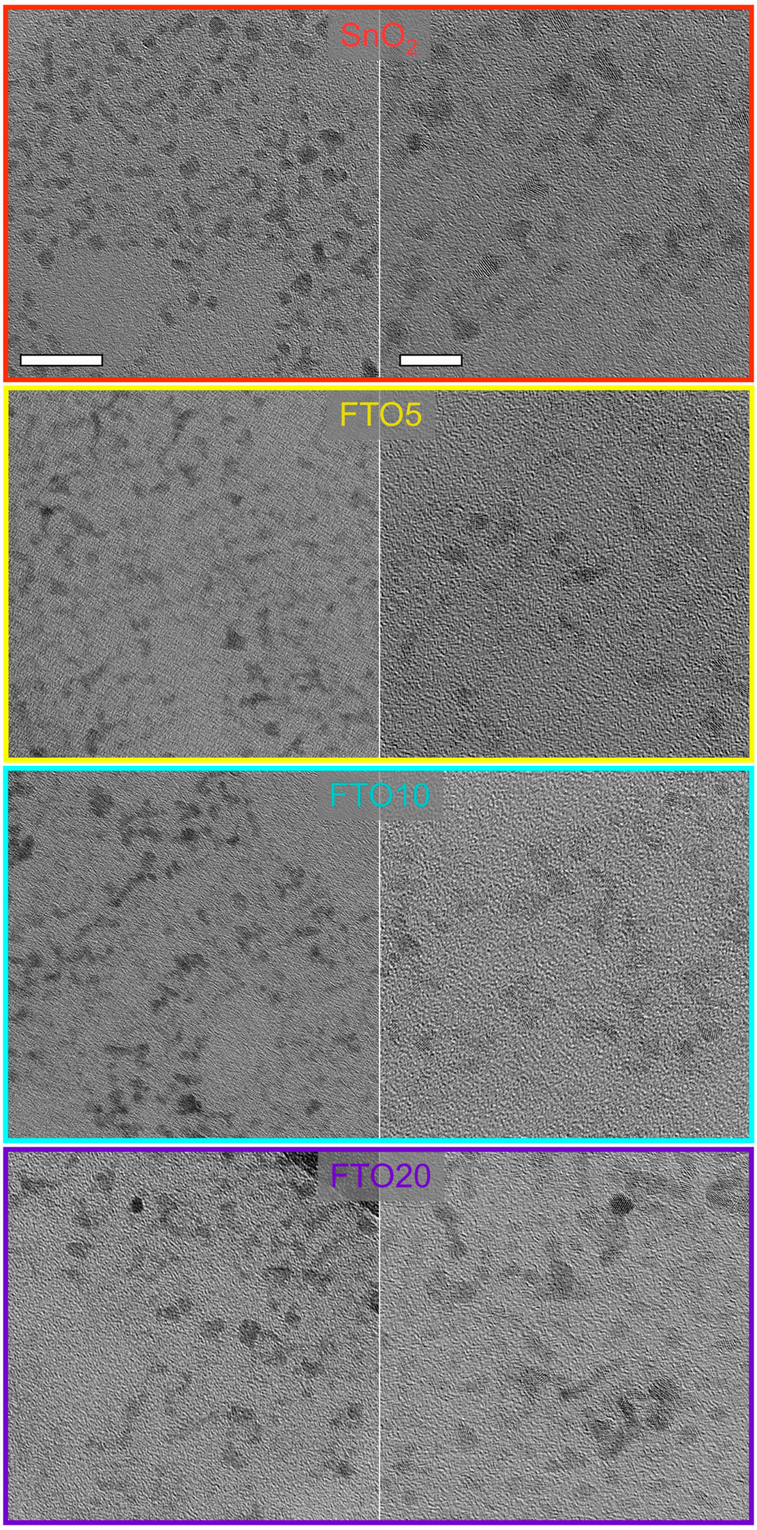


**Figure S3**. Transmission. electron microscopy (TEM) images of doped and undoped $SnO_2$ NCs. The scale bars are 20 nm for the left panels and 10 nm for the right panels.

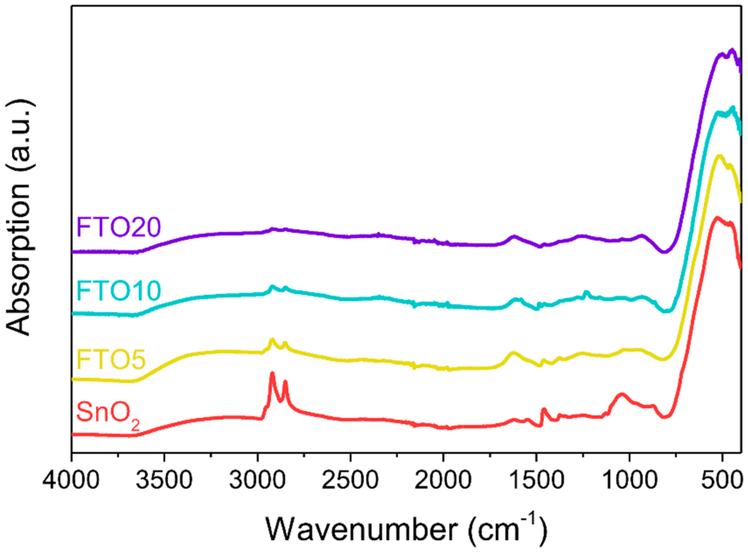


**Figure S4.** Fourier transform infrared (FTIR) spectra for $SnO_2$ and FTO NCs. The vibrational peaks between ~2800–3000 $cm^{-1}$ and at ~1600 $cm^{-1}$ are due to the oleylamine surface ligand. The strong peaks at low wavenumbers (~500 $cm^{-1}$) are due to Sn-O vibrations.

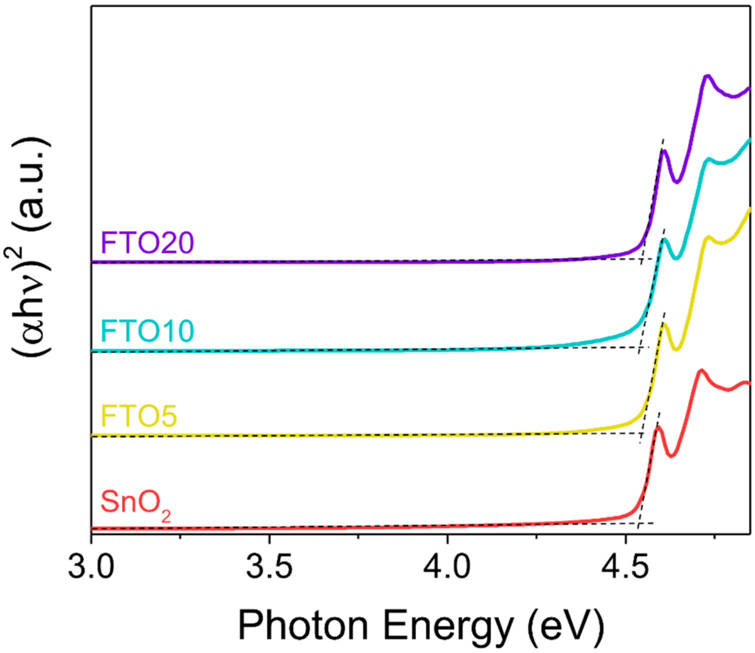


**Figure S5.** Plots obtained from optical absorption spectra of $SnO_2$ and FTO NCs dispersed in chloroform. An optical band gap of ~4.55 eV can be estimated for all samples.

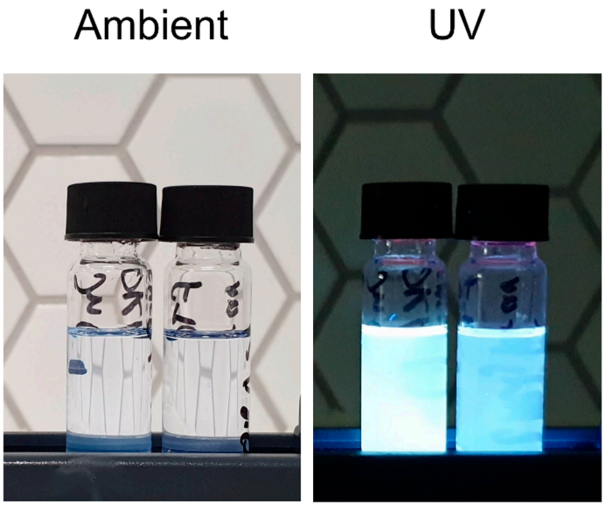


**Figure S6.** Photograph of $SnO_2$ (left vial) and FTO10 (right vial) colloidal solutions exposed to ambient light and UV light. Bright blue luminescence can be seen under UV illumination, while the photo under ambient light shows the perfect dispersion of $SnO_2$ NCs, without any scattering or aggregation phenomena.

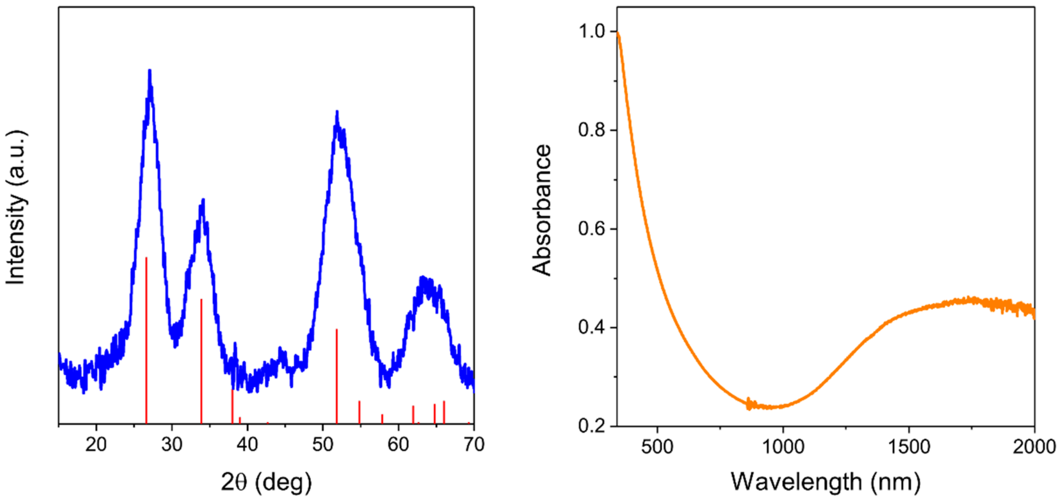


**Figure S7.** Pattern (left panel) and UV-Vis-NIR absorption spectrum (right panel) for Sb-doped tin oxide (ATO) NCs synthesized with the same method employed for FTO NCs, replacing ammonium fluoride with antimony chloride. Phase pure cassiterite $SnO_2$ and a clear LSPR peak in the near infrared can be observed.

We note here that the synthesis of ATO NCs is less reproducible compared to the synthesis of FTO due to the formation of secondary phases and as such will require further optimization. As shown in the XRD patterns below, in certain reactions we observed the formation of metallic antimony (ICDD No. 35-0732, left panel) and sometimes a combination of $SnO_2$, metallic antimony and a SbSn mixed phase (stistaite, ICDD No. 33-0118, right panel).

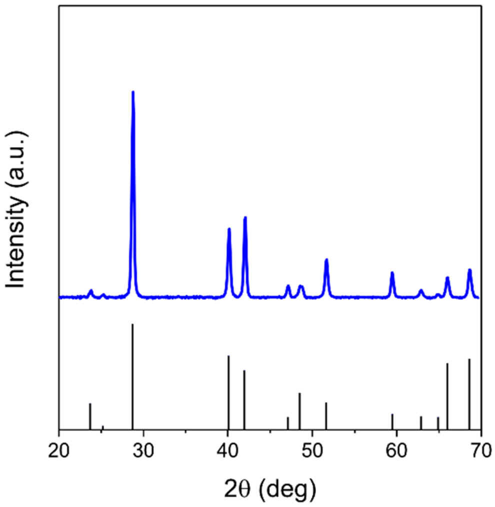
Intensity (a.u.)
20
30
40
50
60
70
2θ (deg)

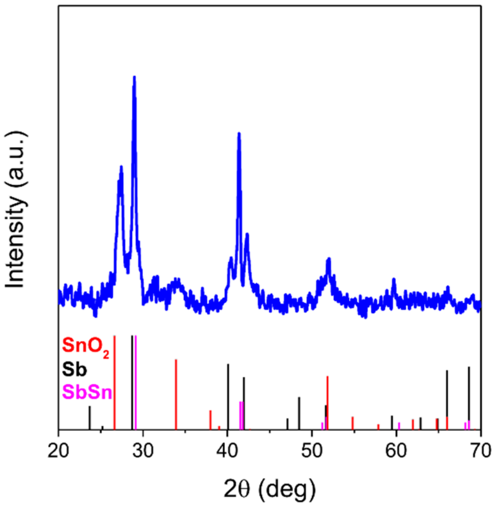
Intensity (a.u.)
SnO2
Sb
SbSn
20
30
40
50
60
70
2θ (deg)
